# A Review on the Wear, Corrosion and High-Temperature Resistant Properties of Wire Arc-Sprayed Fe-Based Coatings

**DOI:** 10.3390/nano11102527

**Published:** 2021-09-27

**Authors:** Joseph Ndiithi Ndumia, Min Kang, Bertrand Vigninou Gbenontin, Jinran Lin, Samuel Mbugua Nyambura

**Affiliations:** College of Engineering, Nanjing Agricultural University, Nanjing 210031, China; 2020212023@stu.njau.edu.cn (J.N.N.); 2019212016@njau.edu.cn (B.V.G.); linjinran@njau.edu.cn (J.L.); accessmbugua@gmail.com (S.M.N.)

**Keywords:** wire arc-spraying, Fe-based coatings, corrosion, wear, high-temperature resistance

## Abstract

Among different thermal spraying methods, arc-spraying has been widely used due to its low operating costs and high deposition efficiency. The rapid progress of cored wire technology in arc-spraying has increased possibilities for the preparation of new Fe-based coating materials with enhanced properties by adding reinforcement particles and alloying elements to suit the different applications. Fe-based coatings have been extensively used because of their high strength, toughness, lower production costs, and availability of raw materials. This makes them suitable replacements for Ni-based coatings in ambient and high-temperature applications. This review discusses the research status and developments of the arc-sprayed Fe-based coatings. The study specifically reviews the wear behavior, corrosion analysis, and high-temperature resistant properties of arc-sprayed Fe-based coatings, aiming to develop an understanding of the protection mechanisms for Fe-based coatings. The performance of the Fe-based coatings depends on the integrity of the coating structure. Optimizing arc-spraying parameters minimizes defects (pores, grain boundaries, unmelted particles, oxides, and microcracks) that deteriorate the coating properties. High amorphous phase content, ceramic reinforcement particles and alloying elements enhance the corrosion, tribological, and high-temperature resistant properties of Fe-based coatings. In high-temperature applications, Fe-based coatings form oxide scales that protect the coating from further oxidation; thus, it is important to select the optimum composition for the alloying elements.

## 1. Introduction

In recent years, thermal spraying-as a surface modification process-produces films and coatings that improve the properties of metal surfaces. Rapid, sustainable economic growth in different countries has provided an opportunity for growth in the thermal spray industry in all aspects, such as the production of feedstock material, spraying systems, and spraying coating contracts [1]. Among different thermal spraying techniques, wire arc-spraying, also referred to as arc-spraying (AS), finds wide applications in the repair and remanufacturing of structural parts in equipment machinery, mining, and power industries [2,3,4,5]. Compared with other thermal spraying methods such as high-velocity oxygen fuel (HVOF) and atmospheric plasma spraying (APS), arc-spraying is preferred due to its relatively lower costs, ease of maintenance, higher production spray rates, and significant economic benefits while spraying over large areas.

New processes and coating materials developed with suitable properties improve the functionality and application of arc-sprayed coatings in different environments. Besides focusing on arc-spraying parameters, developing high-performance and multifunctional coatings drive the flexibility of arc-spraying to achieve coatings with advanced properties [6,7]. Fe-based crystalline and amorphous coatings have great potential in anti-corrosion and anti-wear applications. They are also suitable candidates for medium- to high-temperature applications because of their good oxidation properties. The performance of Fe-based coatings relies on the coating structure, amorphous phase content, and chemical composition of the coatings [8]. The spraying parameters, such as the gas pressure and particle size of the cored powder, affect the coating structure. A dense coating structure with low porosity and grain boundaries enhances the coating performance in ambient and high-temperature conditions. Alloying elements with high glass-forming ability (GFA) can be added to the feedstock material to improve the amorphization of the coating. Ferrous matrices reinforced with particles such as Al_2_O_3_, WC, TiC, and Cr_3_C_2_ improve the tribological and high-temperature properties [9]. Fe-based coatings form oxide scales as protective mechanisms against oxidation and corrosion in high-temperature conditions. Xu et al. [10] elevated the high-temperature properties of FeAl intermetallics by adding abrasive-resistant Cr_3_C_2_. Cr_3_C_2_ in the cored wire improved the coating bond strength and facilitated formation of Cr_2_O_3_ and Al_2_O_3_ oxides, which increased the high-temperature resistant properties. To enhance the high-temperature and corrosion properties, Cr is widely used in Ni-based and Fe-based alloys because it forms the protective Cr_2_O_3_ oxide layer [11]. Ni-based coatings are preferred due to their outstanding properties in high temperatures to prevent sulfidation and oxidation of the substrate. However, due to the high production cost, Fe-based coatings are increasingly being developed by adding alloying elements and ceramic particles to substitute the Ni-based coatings [8,12,13,14]. Fe-based coatings can replace Ni-based coatings because of good mechanical properties, low density, low cost, and availability of raw materials. Li et al. [12] prepared arc-sprayed FeCrSiB and observed that adding Si and B elements reduced the oxide inclusions and increased hardness in the coating compared with NiCrTi and FeCr. Table 1 shows a comparison of properties for arc-sprayed Fe-based and Ni-based coatings. Low porosity, high bonding strength, and hardness enhance the overall properties of Fe-based coatings. Hathaipat et al. [15] showed that arc-sprayed Fe-based alloys offer good intermediate properties as an alternative for the NiAl bond coat at temperatures below 400 °C. The rapid growth of cored wires technology has increased the metallurgical variety of Fe-based coatings. The material composition can be developed to suit the expected performance of the coatings. Fe-based amorphous/nanocrystalline coatings with high metallic glass content, dense structure, and low porosity have been fabricated using arc-spraying [16,17]. The amorphous coatings can be prepared by arc-spraying due to the rapid cooling process of about (~10^5^ Ks^−1^) that prevents crystallization and long-range diffusion [18]. The addition of glass-forming elements (such as B, P, C, Mo, and Si) can also generate a certain amount of amorphous content, which improves the hardness and toughness of the coating [19,20,21].

This review presents the current research status and developments of the arc-sprayed Fe-based coatings. We discussed the wear behavior, corrosion analysis, and high-temperature resistant properties of Fe-based coatings. The protection mechanisms against wear, corrosion, and high-temperature conditions are elaborated.

## 2. Working Principle of the Arc-Spraying Process

**Figure 1:**
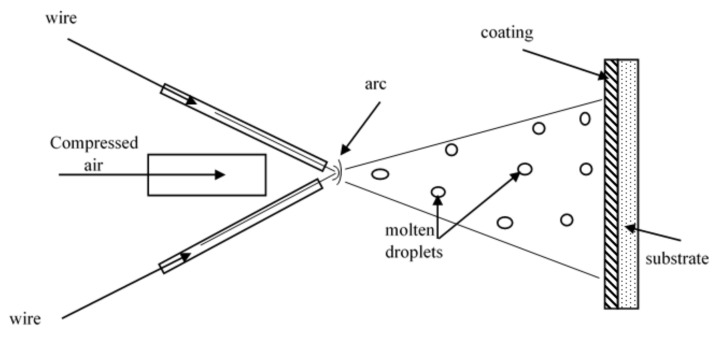
Illustration of the arc-spraying process.

The arc-spraying process melts wires with an electric arc formed at the tip, and the molten or semi-molten materials propelled by a stream of high-pressure velocity gas onto a substrate material to form the coating (Figure 1) [39]. Air, N_2_, and CO_2_ are some of the atomizing gases used in the spraying process. The arc-spraying set-up consists of compressed gas supply, wire feed, arc-spray gun, spray controller, and power supply. The coating formed results from the overlapping splats that interlock and build on top of one another with a cooling rate of ~10^5^ K/s [40]. Molten particles from the electrode tips can reach a temperature of up to 4000–6000 °C; the particles are accelerated toward the substrate surface by an atomizing gas with pressure in the range of 0.27–0.6 MPa; the velocity of particles <300 m/s. Coatings obtained have high adhesion strength of approximately 28–41 MPa and porosity levels of around 3–10%, but this has been improved over time by the formation of amorphous/nanocrystalline coatings and addition of reinforcement particles as shown in Table 1 and Table 2 [39]. In comparison with arc-spraying, high-velocity arc-spraying (HVAS) provides the molten particles with a higher atomizing air pressure and velocity, which improves the quality of the coatings [41,42].

### 2.1. Coating Material Preparation

**Figure 2:**
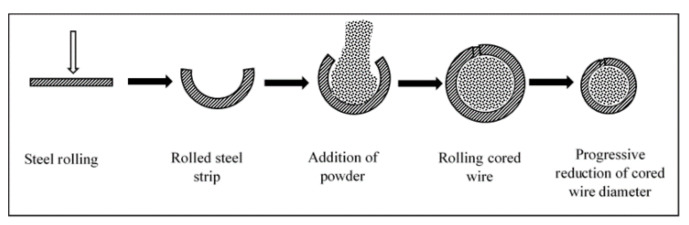
Schematic diagram of cored wire preparation. Reprinted with permission from ref. [44]. Copyright 2007 Elsevier.

The arc-spraying process uses alloy metal wires and cored wires to prepare coatings. Coatings prepared by alloy metallic materials include: CuAl, ZnAl, AlMg, and NiAl. However, alloy wires are limited due to the few ductile materials and the high hardening tendency of metal alloys during the drawing process. Cored wires contain powdered particles inside a sheath of ductile metal or alloy as illustrated in Figure 2. They are beneficial because of their lower cost, shorter production cycle, and combined benefits of the metals and powder materials. They provide diverse options for arc-spraying materials. High hardness materials (oxides, carbides, and nitrides) added into the metal sheath improves the wear and high-temperature resistance of the coatings. The filling factor coefficient, which is the ratio of the core weight to the total weight of the core, determines the material amount in the core. Depending on the diameter of wire, density of core material, and purpose, the filling factor ranges between 15 and 40% [43].

### 2.2. Arc-Sprayed Coating Microstructure

**Figure 3:**
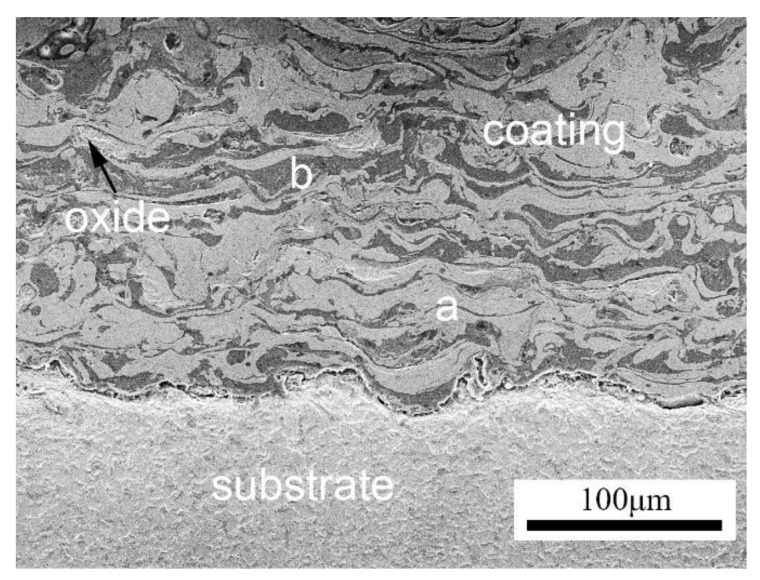
HVAS FeCrAl/Al coating microstructure: (**a**) FeCrAl splats and (**b**) Al splats. Reprinted with permission from ref. [53].

Upon impact on the substrate, molten droplets flatten and solidify to form splats that accumulate and build up the coating. These coatings are characterized by a lamellar structure with alternating layers (Figure 3). Studies have shown the correlation between the operating spray parameters (spray distance, current, arc voltage, and atomizing gas pressure) and the microstructural and mechanical properties of arc-sprayed coatings, hence the need for optimization of spray parameters [45,46,47,48,49]. Pores, oxide inclusions, unmelted particles, and cracks may also be present due to some coating imperfections. A high porosity and oxide content reduce the quality of the coatings. As discussed by Li et al. [50], pores in the coating can be generated as a result of the following: air traps in the molten particles during the solidification process; some splats form zonal fractures along the boundaries because of incomplete accumulation of the flat particles; and, during the solidification of the molten droplets, the density difference between the solid and liquid phases of the droplets leads to volumetric shrinkage. Pores can be beneficial to the coating structure because they reduce the friction during the oil wear test by acting as storage structures for oil. The pores, however, should be kept as minimal as possible. In corrosive environments, pores link the corrosion media to the coating, damaging the underlying coating and substrate. Porosity can be effectively reduced by post-coating techniques such as sealing, heat treatment, and laser remelting [46,51]. Oxides found in the coating have been explained as occurring in three different ways [52]: the primary droplets are oxidized during the atomization process as in-flight particles; the secondary droplets are oxidized from splashing; and the coating surface is oxidized after deposition. In some cases, oxides can be beneficial to the coating properties. As stated by Xu et al. [10], the small oxide particles in the FeAl/Cr_3_C_2_ coating structure enhanced the abrasion resistance, erosion resistance, and bonding strength.

## 3. Properties of Arc-Sprayed Fe-Based Coatings

### 3.1. Wear Properties at Room Temperature

Of the many reasons for material failure, wear causes structural degradation. But, the high hardness, fracture toughness, and good adhesion strength properties increase the wear-resistance of Fe-based alloy coatings in their potential applications. For both wear tests carried out on the pin-on-disc and abrasive wheel testers, the sliding wear behavior of thermal-sprayed coatings could improve by adding hard phases of wear-resistant particulates such as Cr_3_C_2,_ WC, and TiB_2_ into the powder matrix of cored wires, and increasing amorphous phase content in the coating [20,54]. Table 2 summarizes the wear properties of some Fe-based coatings. 

Ding et al. [55] improved the wear properties of arc-sprayed FeCrNi/CBN coatings by adding the hard phase CBN powder during cored wire preparation. The diffused CBN particles presented hard phases, which improved the wear resistance of the coatings by protecting the substrate and controlling the abrasion wear of the coating. Arc-sprayed FeCrBSiNbW coating [25] had an amorphous/nanocrystalline structure with low porosity, dense structure, and high microhardness that improved the wear resistance. The wear resistance of the amorphous coating was better than that of 3Cr13 coating in the same oil-lubricated wear test conditions because of the homogenously dispersed amorphous particles in the glassy phase. The wear resistance increased due to the composite microstructure of the amorphous coating that reduced volume loss attributed to the plasticity and the restriction of cracking of the amorphous phase [21]. Fu et al. [30] compared the wear properties of arc-sprayed Fe-based amorphous coatings filled with NiB and NiB-Cr_3_C_2_ powders. The wear resistance of both coatings was better than that of Q235 steel substrate. The higher wear resistance of NiB-Cr_3_C_2_ was due to the net structure formed by the nanocrystalline particles. The even distribution of the hard boride phase in the metal matrix promoted the high hardness. The wear resistance of NiB was lower than that of NiB-Cr_3_C_2_ coating because of the higher porosity and presence of oxides that formed microcracks under abrasion conditions. In amorphous coatings, the wear resistance of the coatings depends on the coating microstructure and the primary phases. Arc-sprayed Fe-based coating with CrB and Cr_3_C_2_ filler powders were similarly prepared by Fu et al. [31]. The coatings contained rich amorphous phase content with a bit of nanocrystalline structure. The amorphous phase formed due to the high cooling rates of the droplets (~10^5^ K/s) and the high glass-forming ability (GFA) of the composition of cored wires [56]. The amorphous phase transformed into nano crystallites, improving the microhardness. The decreased grain size increases microhardness in nanostructured materials because of the higher density of the coating, which prevents the propagation of dislocations and improves the plastic deformation resistance [57]. Nano crystallites formed during the spraying process due to the amorphous phase devitrifying when substrate temperatures increased. The Fe-based coating had eight times higher relative wear resistance than the commercial 3Cr13 coating. The micro-cutting wear mechanism of the amorphous coating was minimized due to its higher microhardness and the existence of Fe_3_O_4_ lamellae that contributed to the flaking off of the coating. Cheng et al. [58,59] prepared wire arc-sprayed FeBSiCrNbMnY amorphous coatings. Compared with the 3Cr13 coating, the amorphous coatings had a better wear resistance. The dense, compact structure, low porosity, high hardness, and the dispersion strengthening of nanocrystalline grains of the amorphous coating minimized the material removal, hence the excellent wear resistance. The amorphous coating exhibited high hardness to modulus of elasticity ratio (*H/E*), which has a positive influence on the wear properties of the coating [60]. The high wear resistance of the coating was attributed to the effect of the nanocrystalline particles and amorphous phase. The nanocrystalline particles of coatings contain few defects, as stated in [61], hence their high strength and resistance to abrasive wear.

**Table 2:** Summary of the wear and mechanical properties of the arc-sprayed Fe-based coatings.

Coating	Porosity	Hardness (H)	Elastic Modulus (GPa)	*H/E*	Specific Wear Rate	Wear Mechanism
FeCrBSiNbW	2.8%	14.7 GPa (~1499) HV	198	0.074	-	Dispersion strengthening of the amorphous/nanocrystalline grains prevent the material removal [25]
Fe-NiB Fe-NiB-Cr_3_C_2_	2.7% 2.1%	950 HV_0.1_ 1090 HV_0.1_	-	-	-	Wear mechanism of the coatings was by flaking off and some slight plastic furrows [30]
Fe-CrB-Cr_3_C_2_	2.33%	860–1260 HV_0.1_	-	-	-	High hardness prevented micro-cutting. Mass loss by flaking mechanism [31]
FeBSiNb	1.2%	16.42 GPa (~1674) HV	219	0.075		Brittle failure and fracture [62]
FeBSiCrNbMnY	1.7%	15.7 GPa (900–1050) HV_0.1_	217	0.07	-	Brittle failure and fracture [58,59]
3Cr13		6.9 GPa (~704) HV	199	0.035	-	Big pits and parallel grooves characterize cutting and delamination [63]
FePSiBNb	<3%	12.3 GPa (~1254) HV	204	0.06	(0.57 − 1.86) × 10^−5^ mm^3^/Nm (at different loads and sliding speeds)	Oxidative wear coupled with delamination [63]
Fe-FeB-WC/12Co Fe-FeB-WC/12Ni	2.1% 3.2%	920 HV_0.1_ 872 HV_0.1_	-	-	-	Selective removal of the binder caused by plastic deformation and fatigue [64]
FeCrCMoBWSiNb (140MXC)	1.55%	9.1 GPa (~928) HV	-		-	Delamination in combination with plastic deformation and oxidation [21]
FeNiCrBCSi	2.1%	960 HV_0.3_	-	-	-	Selective removal of the binder is probably caused by the plastic deformation and fatigue, Flaking off caused by microcracks [51]
08Mn2Si 4Cr13 65Mn	6.12% 3.33% 5.43%	231.2 HV 288.9 HV 329.9 HV	-	-	-	Abrasive wear [50]
FeCrMnMoWBCSi	4.85%	883.8 HV_0.1_			-	Fatigue wear and oxidation wear [65]
FeCrBSiMnMoW	2.53%	1150 HV_0.3_	-	-	3.3 × 10^−5^ mm^3^/Nm	Abrasive wear mechanism with brittle peeling pit and cracks [66]
FeNiCrAlBRE/Ni95Al	3.74 %	480–600 HV_0.1_	-	-	-	Fracture of splats due to severe plastic deformation at the tip of splats. Cracks initiated at the edges of pores, between the boundaries of inclusions and splats or interfaces of splats [67]
FeNiCrAl/3Cr13	-	375–390 HV_0.1_	-	-	1.963 mm^3^/Nm	Abrasive wear mechanism [33]
WC/W2C-FeCMnSi	5.4% 4.4% 2.7% 2.9% 3.3%	567 ± 63 HV_0.3_ 543 ± 86 HV_0.3_ 561 ± 79 HV_0.3_ 585 ± 117 HV_0.3_ 630 ± 65 HV_0.3_	84.0 81.9 118.3 124.8 151.4	0.057 0.055 0.051 0.042 0.051	-	[45]
FeBSiNbCrMo	1.1%	18.7 GPa (~1907) HV	-	-	-	Brittle delamination [68]
FeBSiNbCr	1.5%	1113 HV	-	-	-	Brittle breaking and fracture [69]

Post-treatment coating techniques such as annealing, surface remelting, and sealing improve the wear resistance of Fe-based coatings. FeNiCrAl coatings were surface-remelted by the tungsten inert gas welding. The wear resistance improved as a result of the reduced pores and cracks. The main abrasive mechanism was cutting and ploughing [70]. The wear resistance of the Fe-based amorphous coatings was enhanced by heat treatment. Fu et al. [51] obtained high wear resistance of the heat-treated Fe-based amorphous coatings due to the increased hardness caused by precipitates that formed during sintering. Yan et al. [65] described the wear properties of arc-sprayed FeCrMnMoWBCSi amorphous alloy coatings after sealing with aluminum phosphate. The sealing agent improved the wear resistance by filling coating defects and hindering crack propagation.

Transition layers or bond coat layers improve the thermal shock resistance and wear resistance by enhancing the bonding strength [71]. FeNiCrAlBRE/Ni95Al was arc-sprayed with Ni95Al applied as a transition layer to improve the adhesive strength between the coating and substrate. The coating had better wear resistance because of the low debris and shallow grooves [67]. Tian et al. [33] reported the abrasive wear properties of the 3Cr13 coating when FeNiCrAl was applied as a transition layer between the coating and the substrate. The low wear volume loss of the composite coating was due to its high hardness caused by evenly distributed Cr_23_C_6_ and (Fe, Cr) phase. The hardness-wear resistance relation could be explained by Archard’s Equation [72], as shown in Equation (1):

(1) $$V_{W}=K\times \frac{SN}{H}$$

where *V_W_* is the worn volume, *K* is the wear coefficient, *S* is the sliding wear distance, *N* is the applied load, and *H* is the hardness.

**Figure 4:**
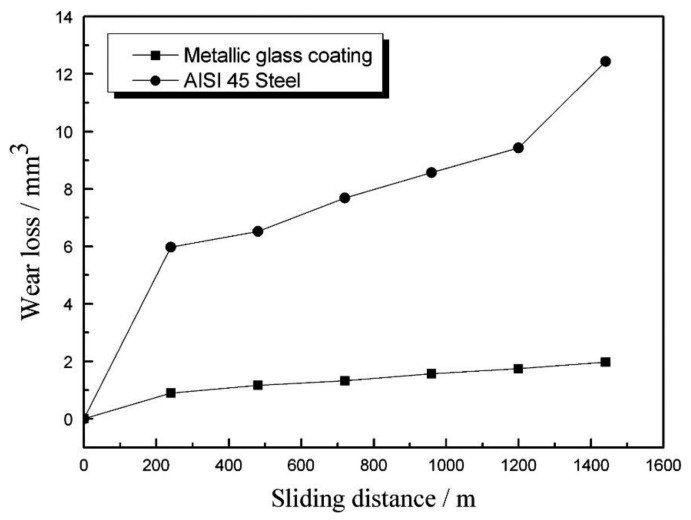
Wear losses of coating and substrate as a function of sliding distance. Reprinted with permission from ref. [74]. Copyright 2013 Elsevier.

According to Equation (1), it can be stated that the high coating hardness contributes to the good wear properties. The wear rate of the coating is proportional to the applied load and sliding distance (sliding time and sliding linear speed) [73]. Volume wear losses of the AISI 45 steel substrate and the metallic glass coatings increased linearly with sliding distance (Figure 4).

**Figure 5:**
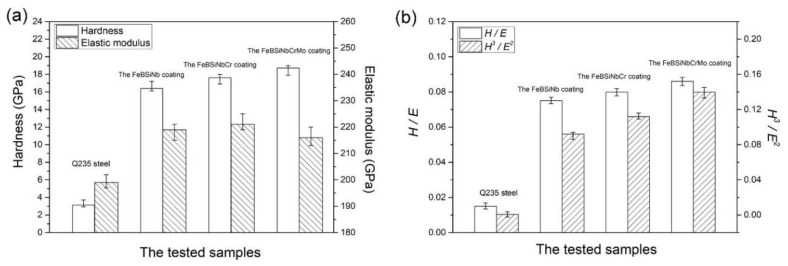
(**a**) Hardness *H* and elastic modulus *E*, (**b**) *H*/*E* and *H^3^*/*E^2^* of the samples. Reprinted with permission from ref. [68]. Copyright 2018 Elsevier.

It is increasingly being recognized that hardness is not the only primary requirement for wear resistance. Researchers have recently examined the effects of elastic modulus on the wear resistance properties. The induced residual stress and mechanical strength of the coating depend on the elastic modulus. The low elastic modulus and high coating hardness contribute to high wear resistance and elastic energy absorption ability [60]. The microhardness and elastic modulus relationship can be derived from the load-displacement curves data by nanoindentation measurement. The ultra-high strength of the amorphous structure and excellent bonding between the elements improves the coating hardness. *H/E* shows the depth of penetration that a coating material tolerates without exceeding the elastic limit. A high hardness to elastic modulus *H/E* ratio indicates good wear resistance of the arc-sprayed coatings. A high *H^3^/E^2^* indicates the resistance of the loaded material to plastic deformation and therefore shows that the material has high toughness [75,76]. Figure 5 shows a relationship of the hardness elastic modulus for the Q235 steel and three metallic glass coatings. The *H/E* and *H^3^/E^2^* increased after addition of Cr and Mo elements promoting lower friction and higher wear resistance [68]. The higher value of *H/E* and *H^3^/E^2^* shows that the ability to absorb applied deformation is strong without exceeding the elastic limit, or adapting to the deformation with less damage [63].

In summary, the wear properties of the Fe-based coatings depend on the coating microstructure, the additional hard reinforcement particles, and the mechanical properties, including hardness and elastic properties. Similarly, the amorphous/nanocrystalline phase structure of the coatings improves wear resistance as a result of the dispersion strengthening effect caused by the homogenous amorphous phase in the glassy state. Further research should be conducted to improve the tribological properties of arc-sprayed Fe-based amorphous coatings by adding reinforcement particles such as WC, Cr_3_C_2_, and Al_2_O_3_.

### 3.2. Corrosion Properties at Room Temperature

Corrosion is among the leading causes of degradation of materials, especially equipment and structures exposed to marine environments. Coating defects such as pores and cracks have adverse impacts on the corrosion properties. They act as passages for the corrosive media, and they should be minimized in the coating microstructure. Lin et al. [26] compared the corrosion properties of arc-sprayed FeB, FeBSi, and FeNiCrBSiNbW coatings. The compact structure with low porosity and amorphous/nanocrystalline structure of the FeNiCrBSiNbW coating offered superior corrosion protection in 3.5 wt. % NaCl solution as summarized in Table 3. The Cr element formed a passivation film, which further enhanced corrosion protection. The absence of the coating defects in the amorphous/nanocrystalline coating improved the corrosion resistance. Pores and cracks resulted in crevice corrosion after exposure to the electrolyte [77]. Arc-sprayed FeCrBSiNbW amorphous coatings displayed better corrosion resistance than 0Cr18Ni9 coatings under the same testing conditions [25]. The structural and chemical homogeneity of the amorphous coating slowed the electrolyte penetration through the coating. The presence of oxides, microcracks, and semi-molten particles in the 0Cr18Ni9 stainless steel coating facilitated the higher corrosion rate.

**Figure 6:**
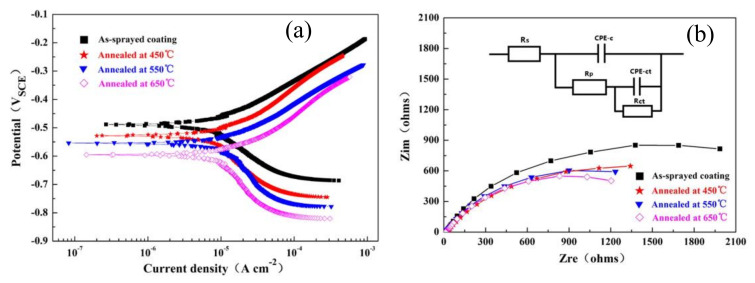
(**a**)Tafel polarization curves and (**b**) fitted EIS data of the as-sprayed and annealed FeNiCrBSiNbW coating. Reprinted with permission from ref. [79]. Copyright 2014 Taylor & Francis.

Post-treatment techniques such as annealing and sealing treatment affect the corrosion properties. Zeng et al. [46] studied the effect of atomizing gas type and sealing treatment on arc-sprayed stainless steel coating. Concerning corrosion properties, nitrogen-atomized coatings were more reliable than air-atomized coatings because of their denser structure, whereas sealed coatings provided better protection to the substrate against corrosion. Annealing amorphous coatings degraded the corrosion resistance due to the increasing amount of crystallization [78]. Annealing FeNiCrBSiNbW coating at different temperatures (450, 550, and 650 °C) reduced the corrosion resistance [79]. With the increase in annealing temperature, the corrosion potentials (*E_corr_*) reduced and the current densities (*I_corr_*) increased, as represented in Figure 6. The electrochemical impedance spectroscopy (EIS) of the as-sprayed coating displayed the largest size of the capacitive semicircle, hence the lowest corrosion rate (Figure 6). The amorphous phase of the coatings transformed into crystalline phase affecting the corrosion resistance. The amorphous phase in the arc-sprayed coating increased corrosion resistance due to the absence of dislocations and grain boundaries. In addition, the Cr element in the arc-sprayed FeNiCrBSiNbW coating formed passive films that improved corrosion resistance.

Cheng et al. [80] studied the effect of crystallization on corrosion performance of arc-sprayed FeBSiNb coatings that were devitrified at different annealing temperatures (500, 550, 600, and 650 °C). The Nyquist plots of the as-sprayed coating showed a larger diameter of the impedance spectra, which decreased with an increasing fraction of crystallization. The lack of defects in the microstructure and chemical homogeneity of the as-sprayed coating resulted in excellent corrosion properties. The crystallization of the amorphous coating led to lattice mismatch and strain, which made the stress caused by the heterogeneity weaken the cohesion between the oxide film and metal, thus causing film rupture. The annealing process caused the generation of structural defects such as intersplat oxides that dissolved forming pores and cracks that allowed electrolyte penetration. Table 3 summarizes the corrosion behavior of arc-sprayed Fe-based coatings.

In summary, the coating microstructure needs to be free of defects such as pores, oxides, unmelted particles, and cracks that accelerate the passage of electrolytes into the underlying coating. Oxides formed during the spraying process also degrade the corrosion resistance by reducing the adhesive strength between the coating splats. These defects can be minimized by optimizing the arc-spraying parameters. Crystallization of amorphous coatings during annealing causes lattice mismatch and strain, which weakens the coherence between the oxide films and metal, leading to the rupture of oxide films [81]. Thus, the annealing temperature and time should be controlled to obtain the optimum properties. Although some research has been carried out on the corrosion behavior of annealed Fe-based amorphous coatings, there is very little discussion about the corrosion behavior of annealed Fe-based crystalline alloys.

### 3.3. High-Temperature Properties of Arc-Sprayed Fe-Based Coatings

High-temperature wear, corrosion, erosion, and oxidation reduce the service life of components used in high-temperature environments. Steam pipes in geothermal power stations and boiler tubes are examples of structures that degrade due to hot corrosion and oxidation conditions. Boiler tubes in thermal power plants are exposed to fly ash particles on impact that result in high-temperature erosion. Arc-sprayed coatings have been applied due to their excellent performance in withstanding corrosion, wear, and erosion at elevated temperatures [37]. Change in the cored wires’ material composition by filling powder containing carbides (WC, Cr_3_C_2_) or oxides (Al_2_O_3_, Cr_2_O_3_) improves the service life of structures under high temperatures.

#### 3.3.1. High-Temperature Oxidation Behavior

From previous works, the coating microstructure, oxidation processes, and phase transformation affect the oxidation resistance of arc-sprayed coatings [8]. Similar to corrosion and wear properties, defects such as coating-substrate delamination, cracks, and porosity are also detrimental to the oxidation resistance. Oxidation resistance in Fe-based coatings has been improved by adding elements such as Cr, Si, Al, which form highly protective oxide scales at high temperatures. The effect of coating imperfections on the high-temperature oxidation of coatings has not been widely researched, and this could be a future topic of interest for arc-sprayed coatings. Li et al. [12] studied the oxidation properties of FeCrB(CSi) coatings with different chromium concentrations. The increase in the Cr content (17, 21, 25 at. %) improved the high-temperature oxidation resistance due to the formation of a protective Cr_2_O_3_ oxide layer that inhibited further oxygen penetration. The coating with the highest amount of Cr (25 at. %) had the least weight gain compared with the uncoated steel substrate and commercial FeCrAl coating. Boron and silicon elements controlled the oxidation of molten particles, thereby preventing Cr consumption. Li et al. [82] also investigated the influence of Cr content (15, 20, 25, 30, 35, 40 at. %) on the high-temperature oxidation properties of Fe-Cr and compared with Ni-Cr-Ti coatings to determine the suitability of Fe-based coatings as substitutes for Ni-based coatings. The Fe-Cr coatings had better high-temperature oxidation resistance than the SA213-T2 substrate used in boiler tubes. The thickness of the oxide scales and final weight gain of the Fe-Cr coatings decreased with an increase in the Cr content at 650 °C for 2 h. The Fe-35Cr and Fe-40Cr had the lowest weight gain rates, showing excellent high-temperature oxidation resistance because of the Cr_2_O_3_ and Fe_2_O_3_ oxide scales that protected the underlying substrate. The high-temperature oxidation resistance of the Fe-35Cr and Fe-40Cr coatings was close to that of the Ni-Cr-Ti coatings.

**Figure 7:**
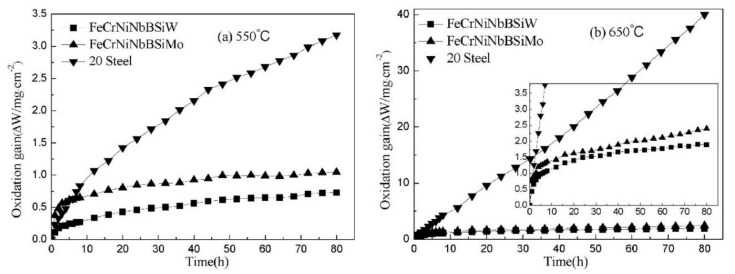
Oxidation kinetics curves of FeCrNiNbBSiW coating, FeCrNiNbBSiMo coating and AISI 20 steel at (**a**) 550 °C and (**b**) 650 °C. Reprinted with permission from ref. [28]. Copyright 2015 Elsevier.

A study by Xin et al. [28] compared the oxidation resistance of arc-sprayed FeCrNiNbBSiW with FeCrNiNbBSiMo. FeCrNiNbBSiW exhibited higher temperature oxidation resistance due to the compact coating microstructure with lower porosity and fewer splashed particles. The oxidized products FeO(Fe, Cr)_2_O_3_ and (Fe, Cr)_2_O_3_/Cr_2_O_3_, listed in Table 4, formed after the temperature increased from 550 °C to 650 °C, acted as protection from further oxidation. Figure 7 shows that the oxidation process of the weight gain curves obeys parabolic law. The weight gains increased rapidly at the initial stage of oxidation and slowed gradually before reaching a stable rate. The parabolic law can be expressed by the following Equation (2) [83]:

(2) $${\left(\Delta W\right)}^{2}=Kt$$

where ∆*W* is the weight gain per unit area (mg/cm^2^), *K* is the constant for oxidation rate, and *t* is the exposure time.

In the study by Luo et al. [84], FeMnCrAl/Cr_3_C_2_ coatings had a higher temperature oxidation resistance than FeMnCr/Cr_3_C_2_ coating, 316L stainless steel coating, and AISI 20 steel. The good coating properties were attributed to the formation of compact Al and (Fe, Cr)-rich oxides that protected the coating from further oxidation. The oxide films, however, were unevenly distributed in the coating because of the porosity and the micro-inhomogeneous composition of the arc-sprayed coatings. Zhang et al. [13] compared high-temperature oxidation properties of HVAS FeCrBAlMo with FeCrBSiMo coatings. The coating with Al had better oxidation resistance than the coating with Si. Al reduced the formation of oxides for other elements and blocked pores in the coating, forming a denser FeCrBAlMo coating structure with higher bonding strength. Alumina and chromium oxides also formed on the surface and densified the coating to protect them from further deterioration.

**Figure 8:**
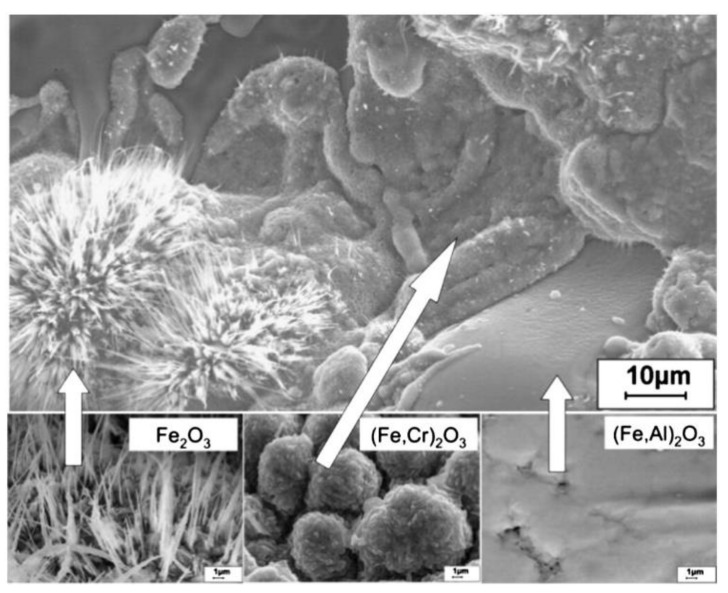
Different morphology of the oxide scales formed on the various lamellae of arc-sprayed coating Cr6B3Al4. Reprinted with permission from ref. [8]. Copyright 2013 Elsevier.

Oxidation tests conducted by Wielage et al. [8] and Vasyl et al. [85] explained the different morphology of oxides formed according to the chemical composition of individual lamellae. Figure 8 shows the oxide films that formed on different lamellae of the arc-sprayed coating [8,85]. The needle-shaped Fe_2_O_3_ formed on the lamellae with low Al and Cr content, monolithic clusters of chromium oxide or alloyed hematite (Fe, Cr)_2_O_3_ formed on the lamellae with higher Cr content, while a dense flat oxide film (Fe, Al)_2_O_3_ formed on the Al-rich lamellae. According to [86], the Fe-based coatings also formed distinctive morphologies depending on their chemical composition. At 800 °C, dense oxide films formed and with increase in temperature to 900 °C, the pores and cracks were filled by a netted oxide scale, which effectively protected the substrate during the long-term exposure.

In conclusion, the high-temperature oxidation of the Fe-based coatings depends on the formation of oxide scales that protect the coatings from further oxidation. Suitable alloying elements such as Cr and Al should be selected and their compositions optimized to enhance the high-temperature oxidation resistance.

#### 3.3.2. High-Temperature Erosion (HTE) Behavior

High-temperature erosion (HTE) has led to the deterioration of turbines, and fluidized bed combustion boilers exposed to particulates flow and fly ash particles. Different factors such as impact angle, impingement velocity, and temperature affect erosion resistance of thermally sprayed coatings. Luo et al. [14] investigated the effects of Al content (0, 8, 15 at. %) on the erosion properties of the FeMnCrAl/Cr_3_C_2_ coating. Sample coatings with 15% Al had the best HTE resistance because of the finer microstructure with low oxide inclusions and pores. The coating without Al had the highest erosion rate under particle impacting. The high amount of oxide fractions weakened the bonding of splats and propagated cracks leading to brittle breaking and lamellar spalling. This study provides a major contribution in the advancement of knowledge on the effects of elemental composition on HTE behavior. Narrow grain size distribution of powders can be applied in arc-spraying cored wires to improve the erosion resistance as used in HVOF thermally sprayed WC-Co-Cr coatings. Narrow powder grain size distribution (36–45 µm) coating gave a higher erosion-corrosion resistance than wider grain size distribution (15–45 µm) because of the different melting behaviors. Overheating of small grains produced phases with lower erosion resistance, hence poor coating quality, while large grains were insufficiently heated, leading to a more porous coating [87]. Nanoparticle coatings produced have higher hardness and toughness as a result of the low porosity and coating defects, which can significantly affect the erosion resistance properties.

The erosion rate of materials at different impact angles can be expressed by Equation (3):

(3) $$Ɛ=Acos^{2}\beta \sin\left(n\beta \right)+Bsin^{2}\beta$$

where Ɛ is the erosion rate, *β* is the impingement angle, *n* is a constant, and *A*, *B* are also constants that describe the brittle and plastic behavior, respectively. For brittle material, *A =* 0, and typical plastic material, *B =* 0, and other material, the plastic materials show the main effects at a low impingement angle while the brittle materials display at a high impingement angle. At high impingement angles, brittle materials have low erosion resistance but they have high erosion resistance at low impingement angle, and it is reversed for plastic materials [88,89].

Xu et al. [10] showed that the impingement angle and the temperature affected the erosion loss of the arc-sprayed Fe-Al/Cr_3_C_2_ coatings. The erosion loss of the coatings decreased with increasing temperature and it was higher at 30° impingement angle than at 90°. During HTE of Fe-Al/Cr_3_C_2_, oxidation films formed, preventing subsequent erosion of particles.

**Figure 9:**
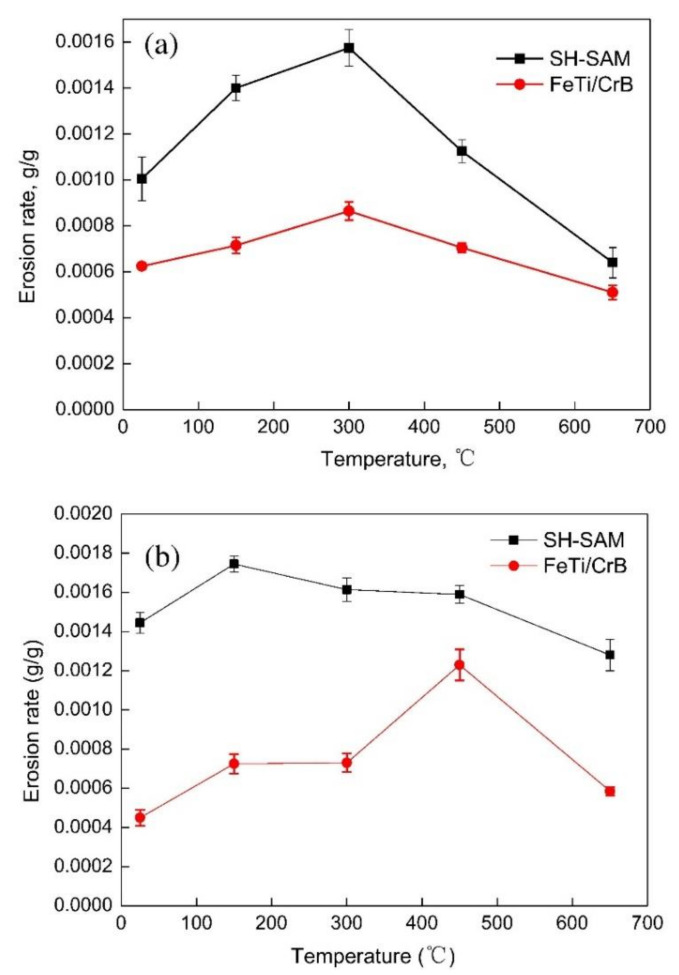
Erosion rates of FeTi/CrB and SH-SAM coatings at (**a**) 30° and (**b**) 90° impact angles. Reprinted with permission from ref. [22]. Copyright 2015 Elsevier.

Yong et al. [22] observed the HTE behavior of arc-sprayed FeTi/CrB coating on 20 g steel substrates under different impact angles and temperatures as shown in Figure 9. The coating had excellent HTE resistance because of its high spalling resistance, high ultimate strength, and toughness. In addition, the CrB, FeB, and oxide films combined into a solid solution which provided good protection against erosion. The FeTi/CrB coating had an excellent erosion resistance compared with the SH-SAM coating. The erosion mechanisms of different coatings are listed in Table 5.

In a comparison study of HVAS Ni-Cr matrix alloy (45PS and SL30) with FeAl/Cr_3_C_2_ coatings [10], the HTE-wear properties were studied using flying ash particles at different temperatures. The erosion rate of all the coatings decreased with an increase in temperature. The surface of the FeAl/Cr_3_C_2_ coating formed Cr_2_O_3_, FeCr_2_O_4_, and Al_2_O_3_ oxides while the Ni-Cr alloys formed NiO, Cr_2_O_3_, and NiCr_2_O_4_ oxides that protected the coatings from further oxidation at 550 °C. Cheng et al. [89] discussed the erosion resistance of arc-sprayed FeBSiNb amorphous coatings at different impact angles, temperatures, and velocities. The erosion resistance of the coating was attributed to the following: as the temperature increased, more nanoscale grains formed, improving the coating microhardness and preventing material removal; thick oxide scales formed and erosion took place on the formed oxide scale; oxidation of the coating and eroded ash particles led to weight gain, which embedded on the coating surface, hence reduction in the erosion rates. The erosion rate of FeBSiNb increased with increasing velocity and followed the empirical power law of erosion given by Equation (4):

(4) $$Ɛ=Kv^{n}$$

where Ɛ is the erosion rate, *K* is a constant, *v* is the velocity, and *n* has values between 2.2 and 3.0 [90].

Future research could focus on the effects of compositions of different alloying elements on high-temperature erosion resistance of arc-sprayed coatings.

#### 3.3.3. High-Temperature Corrosion Behavior

Hot corrosion generally refers to the accelerated oxidation of metals and alloys at intermediate temperatures (600–850 °C) associated with mixed molten salts (e.g., sulphates deposited by alkali metals). Few studies on the high-temperature corrosion properties have been reported and further research on the effect of elemental compositions could be undertaken. Shukla et al. [93] fabricated arc-sprayed FeCrBSiMn coating with superior high-temperature corrosion resistance exposed to a molten salt environment (Na_2_SO_4_ − 82%Fe_2_(SO_4_)_3_) at 900 °C under cyclic conditions for 50 cycles. They noted that the oxides of Fe and Cr enhanced the corrosion resistance of the coatings. Xu et al. [10] prepared HVAS Fe-Al/Cr_3_C_2_ coatings on 20-grade steel. The coatings had a lower corrosion rate than the substrate. The Fe-Al matrix composites and Cr_2_C_3_ had good corrosion properties and the oxides formed protected the coatings. The corrosion rate increased with temperature but later slowed down with increased time. Cr_2_O_3_ facilitated the formation of Al_2_O_3_ that further protected the coating. Guo et al. [94] studied the hot corrosion resistance of FeCrBC in a mixed solution of Na_2_SO_4_ + K_2_SO_4_ (7:3) at 700 °C for 156 h. Thin films of Cr_2_O_3_ formed on the coating and separated the alloy from the mixed salt solution, thus providing better resistance to hot corrosion. In a comparison study by Li et al. [11], the arc-sprayed FeCrSiB coatings provided better hot corrosion resistance than FeCr, approaching that of NiCrTi coating. The weight gain for the hot corrosion has been calculated as follows:

(5) $$\Delta W_{i}=\left[\frac{W_{i+2}-W_{i}}{A}\right]-\left[\frac{W_{i+1}-W_{i}}{A}\right]\times 0.6$$

where, *W_i_* is the mass of the sample before the *_i_^th^* corrosion, *W_i+_*_1_ is the mass after the first salt coating, *W_i+_*_2_ is the mass after the *_i_^th^* corrosion, *A* is the total surface area of the sample, and 0.6 is the coefficient for deducting the salt film crystal water.

The microstructure and chemical composition of the coating material is equally as important and interlayer pores can act as channels for molten salts degrading the underlying coating. Therefore, the coating should be sprayed carefully to avoid penetration of molten salts into the coating [95]. Table 6 summarizes the hot corrosion behavior of the Fe-based coatings.

In the study of hot corrosion behavior, the coating porosity is the key to reducing the corrosion rate. Dense coatings have a better hot corrosion resistance than porous coatings. Corrosion attack on the grain boundaries occurs because of the slipping of the corrosive medium into the substrate [96]. Compared with their crystalline counterparts, most Fe-based amorphous coatings show better corrosion resistance because they lack grain boundaries, cracks, and interfacial defects. Hot corrosion behavior could be used to further explore the corrosion mechanisms of arc-sprayed Fe-based amorphous coatings at elevated temperature.

#### 3.3.4. High-Temperature Wear Behavior

Several studies have shown the relation between the coating integrity and the coating’s high-temperature wear performance. Stress concentration occurs at the defects such as cracks on the coating, which propagate coating failure during the wear process [98]. The coating hardness, cohesive strength of the coating, and bond strength of the coating with substrate influence the high-temperature wear properties. 

**Figure 10:**
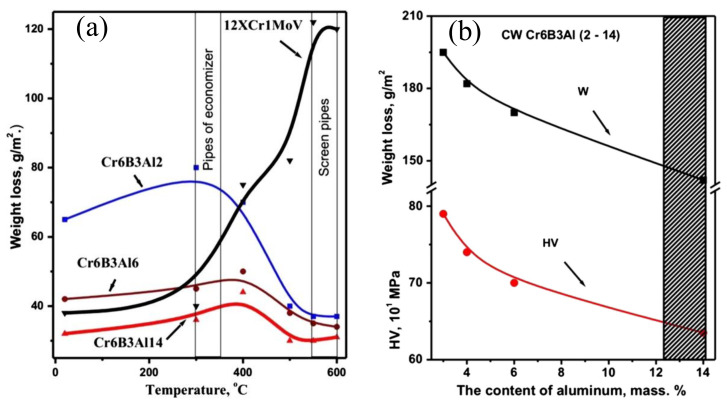
(**a**) Hot gas abrasive resistance of arc-sprayed coatings with different Al content. (**b**) Effect of Al content in the cored wires on the weight loss and microhardness during high-temperature abrasive wear test. Reprinted with permission from ref. [8]. Copyright 2013 Elsevier.

A study on FeAl intermetallic coatings [99] showed a low wear rate at elevated temperatures of up to 650 °C. The oxide films protected the worn surfaces by minimizing the wear rate. Additionally, the high strength and coating hardness resisted further propagation of crack failure and fracture of the coating at high temperatures. FeAl intermetallic coatings displayed high-temperature oxidation resistance by adding Cr_3_C_2_, which improves the abrasion resistance. The coatings had a high wear resistance at room temperature, which decreased with increasing temperature. High hardness and formation of Al_2_O_3_ and Cr_2_O_3_ oxides maintained a higher wear resistance than that of the 20-grade steel. At high temperatures, peeling was the primary wear behavior for the HVAS Fe-Al/Cr_3_C_2_ coatings [10]. Zhu et al. [100] studied the friction and wear behavior of Fe-Al/WC intermetallic coating at temperatures of 650 °C. During the wear process, a protective oxide layer formed, providing solid lubrication and reducing direct contact with the friction counterpart material. The high-temperature strength and high-temperature hardness prevented the propagation of cracks and fracture of particles in the process of sliding wear. Wielage et al. [8] showed that the amount of Al content (2, 6, 14 wt. %) on the hot gas abrasive wear affected the wear loss and microhardness of the arc-sprayed coatings. At temperatures of up to 600 °C, arc-sprayed FeCrBAl coatings with 2 wt. % and 6 wt. % Al content had poorer wear resistance than steel substrate but coatings with 14 wt. % Al content had better wear resistance (Figure 10). Figure 10a,b show the influence of Al content on the weight loss and hardness during the high-temperature abrasive test. The coating with high Al content had improved wear resistance due to the increased coating heterogeneity and the reduced tensile stresses as a result of the oxidation of microcracks and oxidation of the lamellae. Therefore, the hot gas abrasive wear resistance of the coatings depends on the chemical composition of the arc-sprayed coating, residual stresses in the coating, and the chemical homogeneity of element distribution. These factors determine the oxidation intensity in the coating exposed to high temperatures. Precipitation phases formed at high temperatures strengthen the coating to improve the wear resistance. Table 7 summarizes the wear mechanisms of Fe-based coatings at high temperatures.

In conclusion, the phase composition of the coatings and the oxides formation controlled the high-temperature wear, oxidation, corrosion, and erosion resistance. The oxide scales generated stresses that determined the spalling and cracking of the coatings. Adding alloying elements such as Al and Cr is vital to form oxides that reduce the wear and corrosion rate at high temperatures. The coating defects undermine the coating protection at high temperatures, hence the need to minimize them through process optimization. The high-temperature wear resistance has mostly been characterized by the weight gain and weight reduction of the coatings. The degradation of coatings in corrosive medium at high temperature involves the combination of wear process and the electrochemical corrosion process. The corrosion-wear behavior of arc-sprayed Fe-based coatings should be investigated at high temperatures to further understand the tribological properties.

## 4. Conclusions and Future Scope Recommendations

Arc-spraying is a reliable and effective technique used in the production of Fe-based coatings. Arc-sprayed Fe-based coatings are recommended for application in boilers, steam pipes, and components operating at high temperatures due to the high resistance offered with respect to oxidation, corrosion, wear, and erosion. Fe-based coatings have potential to be used as alternatives to some of the Ni-based coatings because of their relatively lower costs and availability of raw materials. Some Ni-based coatings produce oxidation products such as NiO and NiCl_2_, which may be harmful to human health [82]. The protective mechanisms of Fe-based coatings were discussed and the following conclusions and recommendations are made:

1. The density, size, and structure of feedstock powders influence the phase composition of the deposited coatings in HVOF and APS thermal spraying methods. Cored wires in arc-spraying can explore the use of different-sized powders as filling materials to optimize the coating properties. Coating powders of arc-sprayed cored wires can apply nanoscale particles that result in densely packed nanostructured coatings [57]. Arc-sprayed FePSiNb coatings exhibited a nanoscale structure with a grain size range from 12 to 50 nm with good wear resistance properties [63]. More work will need to be done to determine the production and study of properties of arc-sprayed nanostructured coatings.
2. The spraying parameters play an important role in determining the microstructural properties of the coatings. Optimizing methods such as response surface methodology (RSM) analyzes the interaction between spray parameters and their influence on the coating properties. The effects of process parameters on the amorphization of the arc-sprayed coatings could be studied to maximize the amorphous content of Fe-based amorphous coatings.
3. The arc-sprayed Fe-based coatings have better hardness and wear resistance properties than conventional alloys due to the dense microstructure, the dispersion strengthening of the amorphous/nanocrystalline phases, and reinforcement ceramic particles. The elastic properties also determine the wear resistance of the Fe-based coatings.
4. To increase the corrosion resistance, the coating defects (oxides, pores, and cracks) in the Fe-based coatings should be minimized by optimizing spray parameters to prevent the deterioration of coating properties in corrosive media.
5. The high-temperature properties of the Fe-based coating are mainly affected by the microstructure and the elemental composition. The reinforcement ceramic particles added to the Fe-based alloys improve the tribological and high-temperature coating performance while the amorphous phase content is characterized by fewer dislocations, microcracks, and grain boundaries enhancing the properties of the Fe-based amorphous coatings. Future research should focus on understanding the combined corrosion-wear behavior of arc-sprayed Fe-based coatings at elevated temperatures.
6. Adding appropriate alloying elements such as Al and Cr to Fe-based coatings improves high-temperature protection by forming oxide scales that prevent further oxidation of the underlying substrate. Future research should investigate the influence of different elements on high-temperature properties of Fe-based coatings.

## Figures and Tables

**Table 1:** Comparison of properties for arc-sprayed Ni-based and Fe-based coatings.

**Fe-Based Coatings**		**Coating Properties**		**References**
	**Hardness**	**Porosity**	**Bond Strength**	
FeTi/CrB	62.7 HRC (~805 HV)	2.7%	40.21 MPa	[22]
FeCrB	811.4 HV_0.1_–920.1 HV_0.1_	3.31%–4.01%	-	[23]
FeNiBCrSi	700–1025 HV_0.1_	-	57 MPa	[24]
FeCrBSiNbW	14.7 GPa (~1499 HV)	2.8%	-	[25]
FeNiCrBSiNbW	850–1000 HV_0.1_	1.8%	52.1 MPa	[26,27]
FeCrNiNbBSiMo	-	3.46%	42.3 MPa	[28]
FeAl	6.47 GPa (~659.7 HV)	1.83%	24.5 MPa	[29]
FeNiB-Cr_3_C_2_	1090 HV_0.1_	2.1%	-	[30]
FeCrB-Cr_3_C_2_	860–1260 HV_0.1_	2.33%	-	[31]
FeCr	480 HV_0.1_	5.02%	-	[11]
FeCrSiB	650 HV_0.1_	4.08%	-	[11]
FeNiCrAl	626 HV_0.1_	8.76%	52.3 MPa	[32]
3Cr13/FeNiCrAl	375–390 HV_0.1_	-	45.7 MPa	[33]
Fe-Cr-B-C	6.47 GPa (~659.7 HV)		-	
**Ni-Based Coatings**		**Coating Properties**		
	**Hardness**	**Porosity**	**Bond Strength**	**References**
FeCrAl/Ni95Al	530 HV_0.1_	-	43 MPa	[34]
NiCrMoAl	3.65 ± 0.56 GPa (~372.2 HV)	2.4%	-	[35]
Ni-5Al	290 HV	<2%		[36]
Ni-5Al	203.8 HV & 249 HV	1.55%–1.58%	-	[37]
Ni-20Cr	273.5 HV & 379.8 HV	1.53%–1.54%	-	[37]
NiCrTi	380 HV_0.1_	2.49%	-	[11]
Ni-30Cr	244 ± 12 HV_0.3_	8.4%	-	[38]
Ni-45Cr	242 ± 11 HV_0.3_	5.0%		
Ni-50Cr	209 ± 7 HV_0.3_	6.1%		

**Table 3:** Summary of the corrosion properties of Fe-based coatings.

Coatings	Substrate	Current Densities *I_corr_* (µA/cm^2^)	Current Potential *E_corr_* (V)	Corrosion Behavior
0Cr18Ni9 FeCrBSiNbW	AISI 1045 Steel	32.6 4.3	−0.68 −0.45	Chemical and structural homogeneities of the amorphous coating with a dense structure and low porosity prevent electrolyte penetration. Absence of defects, grain boundaries, precipitates, and segregation. The ability of Cr to form a protective film [25].
FeB FeBSi FeNiCrBSiNbW	AISI 1045 steel	18.96 12.69 8.72	−0.758 −0.738 −0.447	Dissolution of Cr to produce a rich passive film, homogenous amorphous/nanocrystalline, compact structure with low porosity and low oxide content which improved corrosion resistance of the coating [26].
FeBSiNb FeBSiNbCr FeBSiNbCrMo	Q235 Steel	5.92 3.61 1.53	−0.847 −0.802 −0.775	Corrosion resistance is attributed to its glassy structure and chemical compositions. The lower porosity and the formation of chromium-rich oxide and Mo-rich passive film. Addition of Mo facilitates the passivation of Cr films [68].

**Table 4:** Summary of the high-temperature oxidation behavior of arc-sprayed Fe-based coatings.

Coatings	High-Temperature Oxidation Data		
	Temperature (°C)	Oxidation Weight Change	Oxidation Products
FeCrAl Fe_17_CrB(CSi) Fe_21_CrB(CSi) Fe_25_CrB(CSi)	650	-	Fe_2_O_3_, Cr_2_O_3_, Al_2_O_3_ Fe_2_O_3_, Cr_2_O_3_, CrBO_3_ [12]
Fe-15Cr, Fe-20Cr, Fe-25Cr Fe-30Cr Fe-35Cr, Fe-40Cr	650	-	Fe_2_O_3,_ Fe-Cr-O spinel Cr_2_O_3,_ Fe_2_O_3_, Fe-Cr-O Fe_2_O_3_, Cr_2_O_3_ [82]
FeCrNiNbBSiW FeCrNiNbBSiMo	550 and 650	-	FeO.(Fe, Cr)_2_O_3_, (Fe, Cr) _2_O_3_, (Fe, Cr)_2_O_3_/Cr_2_O_3_ [28]
FeMnCr/Cr_3_C_2_ FeMnCrAl/Cr_3_C_2_	700	11.209 mg/cm^2^ 4.369 mg/cm^2^	Fe_2_O_3_, Cr_2_O_3_, Al_2_O_3_ [85]
FeCrBSiMo	550 and 650	-	Fe_2_O_3_/FeO.(Fe,Cr)_2_O_3_, (Fe,Cr) _2_O_3_ [13]
FeCrBAlMo	550 and 650	-	Fe_2_O_3_, FeO.(Fe, Cr)_2_O_3_, (Fe,Cr) _2_O_3_, AlFeO_3_ [13]
FeMnCrNiAl/Cr_3_C_2_	800	-	Fe_2_O_3_, Cr_2_O_3_, Al_2_O_3_ [84]
FeCrBAl	600–700	12–20 g/m^2^.h	(Fe, Cr)_2_O_3_, Fe_2_O_3_, (Fe, Al)_2_O_3_ [8]

**Table 5:** Summary of the high-temperature erosion behavior of arc-sprayed Fe-based coatings.

Coatings	High-Temperature Erosion		
	Temperature °C	Impact Angle	Erosion Behavior
Alpha 1800	Room temperature (RT), 300, 400, 500, 600	30° and 90°	Erosion damage was by extrusion-forging mechanism. Shallow craters formed by particle impact and subsequent impact forged platelets into the surface [91]
FeBSiNb	300, 450, 600	30° and 90°	Lower erosion rate at impact angle of 30° and erosion rate decreased with increasing temperature. Mass loss attributed to splat flaking. Main failure mechanism was brittle fracture [89]
FeAl/Cr_3_C_2_	550, 650, 800	90°	Erosion rates decreased with increasing temperature. Fe_2_O_3_, Al_2_O_3_, and Cr_2_O_3_ oxides formed protection coating from further oxidation [10]
FeCrBSiMnNbY FeBSiNbCr	25, 300, 450, 650	30° and 90°	Erosion rates decreased with increasing temperature. FeBSiNbCr had better corrosion resistance than FeCrBSiMnNbY. Erosion mechanism is the brittle breaking and fracture mechanism [92]
FeTi/CrB	20, 150, 300, 450, 650	30°, 60°, and 90°	Erosion rate increased with increasing impact angle. Abrasive cutting and plough wearing were the main erosion mechanisms [22]
FeMnCr/Cr_3_C_2_ FeMnCr8Al/Cr_3_C_2_ FeMnCr15Al/Cr_3_C_2_	900	30°, 45°, 60°, and 90°	Erosion mechanism was through brittle breaking, cutting, and fatigue spalling [14]

**Table 6:** Summary of the hot corrosion behavior of arc-sprayed Fe-based coatings.

Coatings	Corrosive Environment	Temperature (°C)	Hot Corrosion Behavior
FeCrSiB	Na_2_SO_4_ − 25% K_2_SO_4_	650	Formation of Cr_2_O_3_ and SiO_2_ mixed scale with low diffusion coefficients preventing further penetration of solution [11].
FeCrBSiMn	Na_2_SO_4_ − 82% Fe_2_(SO_4_)_3_	900	Fe and Cr enhanced the corrosion resistance of the coatings [93].
Fe-Al/Cr_3_C_2_	Na_2_SO_4_ + K_2_SO_4_ (7:3)	450, 650, 800	The formation of Cr_2_O_3_ oxides speeded the formation of Al_2_O_3_, which protected the coatings [10].
FeCrBC	Na_2_SO_4_ + K_2_SO_4_ (7:3)	700	Compact and dense Cr_2_O_3_ acted as diffusion barriers for the solution [94].
FeNiCr/Cr_3_C_2_	Na_2_SO_4_ + K_2_SO_4_ (7:3)	700	Oxidation, sulfidation, and internal sulfidation were the main hot corrosion mechanisms [97]

**Table 7:** Summary of the high-temperature wear behavior of arc-sprayed Fe-based coatings.

Coatings	Temperature	High-Temperature Wear Behavior
FeAl	Up to 650 °C	Coefficient of friction decreased with temperature increase and protective film formed during the sliding process. Delamination was the main wear mechanism. High strength and hardness Fe_3_Al and FeAl intermetallics prevented crack propagation and fracture of splats [99].
FeAl/Cr_3_C_2_	Up to 600 °C	Main wear mechanism was peeling wear. The Cr_2_O_3_ facilitated the formation of Al_2_O_3_ to reduce the wear loss, High hardness and the good amalgamation between the Cr_3_C_2_ and FeAl intermetallics improved the coating ductility [10].
FeAl/WC	Up to 650 °C	Main wear mechanism was delamination. Coefficient of friction decreased due to oxide films that acted as a solid lubricant during sliding wear [100].
FeCrBAl	600 °C	Increased Al content improved the wear resistance. Lower tensile stresses formed as the coating heterogeneity increased with an increase in Al content. Reduction of tensile stresses was due to the oxidation of microcracks and coating lamellae [8].
